# Biosynthesis and biological action of pineal allopregnanolone

**DOI:** 10.3389/fncel.2014.00118

**Published:** 2014-05-05

**Authors:** Kazuyoshi Tsutsui, Shogo Haraguchi

**Affiliations:** Laboratory of Integrative Brain Sciences, Department of Biology and Center for Medical Life Science, Waseda UniversityTokyo, Japan

**Keywords:** neurosteroids, allopregnanolone, caspase-3, apoptosis, cell survival, pineal gland, Purkinje cell

## Abstract

The pineal gland transduces photoperiodic changes to the neuroendocrine system by rhythmic secretion of melatonin. We recently provided new evidence that the pineal gland is a major neurosteroidogenic organ and actively produces a variety of neurosteroids *de novo* from cholesterol in birds. Notably, allopregnanolone is a major pineal neurosteroid that is far more actively produced in the pineal gland than the brain and secreted by the pineal gland in juvenile birds. Subsequently, we have demonstrated the biological action of pineal allopregnanolone on Purkinje cells in the cerebellum during development in juvenile birds. Pinealectomy (Px) induces apoptosis of Purkinje cells, whereas allopregnanolone administration to Px chicks prevents cell death. Furthermore, Px increases the number of Purkinje cells that express active caspase-3, a crucial mediator of apoptosis, and allopregnanolone administration to Px chicks decreases the number of Purkinje cells expressing active caspase-3. It thus appears that pineal allopregnanolone prevents cell death of Purkinje cells by suppressing the activity of caspase-3 during development. This paper highlights new aspects of the biosynthesis and biological action of pineal allopregnanolone.

## Introduction

*De novo* formation of neurosteroids in the brain was originally demonstrated in mammals (Corpéchot et al., 1981, 1983; Robel and Baulieu, 1985; Lanthier and Patwardhan, 1986; Robel et al., 1987; Jo et al., 1989; Mathur et al., 1993; Mellon and Deschepper, 1993; Compagnone et al., 1995; Ukena et al., 1998, 1999; Sakamoto et al., 2001b, 2003a), and subsequently in non-mammalian vertebrates, such as birds, amphibians, and fish (Mensah-Nyagan et al., 1994, 1996a,b, 1999; Tsutsui and Yamazaki, 1995; Usui et al., 1995; Vanson et al., 1996; Tsutsui et al., 1997, 1999, 2003a, 2008; Beaujean et al., 1999; Schlinger et al., 1999; Takase et al., 1999, 2002, 2011; Freking et al., 2000; Matsunaga et al., 2001, 2002, 2004a; Sakamoto et al., 2001a; Tsutsui and Schlinger, 2001; Ukena et al., 2001; Inai et al., 2003; London et al., 2003, 2006, 2010; Soma et al., 2004; Menuet et al., 2005; Do-Rego et al., 2007; London and Schlinger, 2007; Tam and Schlinger, 2007; Bruzzone et al., 2010; Haraguchi et al., 2010, 2012a; Diotel et al., 2011; Brion et al., 2012). Therefore, *de novo* synthesis of neurosteroids from cholesterol is considered to be a conserved property in the brain across vertebrate species (for reviews, see Baulieu, 1997; Tsutsui et al., 1999, 2000, 2003a, 2006; Compagnone and Mellon, 2000; Mellon and Vaudry, 2001; Tsutsui and Mellon, 2006; Do-Rego et al., 2009).

Until recently, it was generally accepted that neurosteroids are produced in glial cells and neurons which are located in the brain and peripheral nervous systems (for reviews, see Baulieu, 1997; Tsutsui et al., 1999, 2000, 2003a, 2006; Compagnone and Mellon, 2000; Mellon and Vaudry, 2001; Tsutsui and Mellon, 2006; Do-Rego et al., 2009). However, we recently discovered that the pineal gland actively produces neurosteroids *de novo* from cholesterol in the juvenile chicken and quail (Hatori et al., 2011; Haraguchi et al., 2012b). Notably, allopregnanolone (3α,5α-tetrahydroprogesterone; 3α,5α-THP) is a major neurosteroid produced in the pineal gland (Haraguchi et al., 2012b). Importantly, allopregnanolone secreted by the pineal gland prevents cell death of Purkinje cells by suppressing the activity of caspase-3, a crucial mediator of apoptosis, in the cerebellum during development (Haraguchi et al., 2012b).

## Neurosteroidogenic cells in the brain

Past studies demonstrated that oligodendrocytes are the primary site for neurosteroid formation in the brain (for reviews, see Baulieu, 1997; Compagnone and Mellon, 2000). Subsequently, astrocytes were shown to express steroidogenic enzymes (Mellon and Deschepper, 1993). Based on extensive studies, it was generally accepted that glial cells are the site for neurosteroid formation in the brain. However, whether neurons located in the brain produce neurosteroids was unknown in vertebrates until the middle 1990s. We discovered that Purkinje cells, a major neuronal population actively produce a variety of neurosteroids *de novo* from cholesterol in the brain of various vertebrates (Tsutsui and Yamazaki, 1995; Usui et al., 1995; Ukena et al., 1998, 1999; Takase et al., 1999; Matsunaga et al., 2001; Sakamoto et al., 2001a,b, 2003a; Agís-Balboa et al., 2006, 2007). The Purkinje cell expresses several kinds of key steroidogenic enzymes in rat (Furukawa et al., 1998; Ukena et al., 1998, 1999; Sakamoto et al., 2003a). In the rat hippocampus, the expression of steroidogenic enzymes has also been found in pyramidal neurons in the CA1-CA3 regions as well as granule cells in the dentate gyrus (Kimoto et al., 2001; Hojo et al., 2004; Okamoto et al., 2012). In addition to these brain neurons, the expression of steroidogenic enzymes has been reported in neurons in the retinal ganglion, sensory neurons in the dorsal root ganglia and motor neurons in the spinal cord of rat (Guarneri et al., 1994; Compagnone et al., 1995). Based on these findings, not only glial cells but also neurons have been demonstrated as the sites of neurosteroid formation in the central and peripheral nervous systems (for reviews, see Baulieu, 1997; Tsutsui et al., 1999, 2000, 2003a, 2006; Compagnone and Mellon, 2000; Mellon and Vaudry, 2001; Tsutsui and Mellon, 2006; Do-Rego et al., 2009).

## Biosynthesis of neurosteroids in the pineal gland

### Neurosteroids formed in the pineal gland

**Figure 1:**
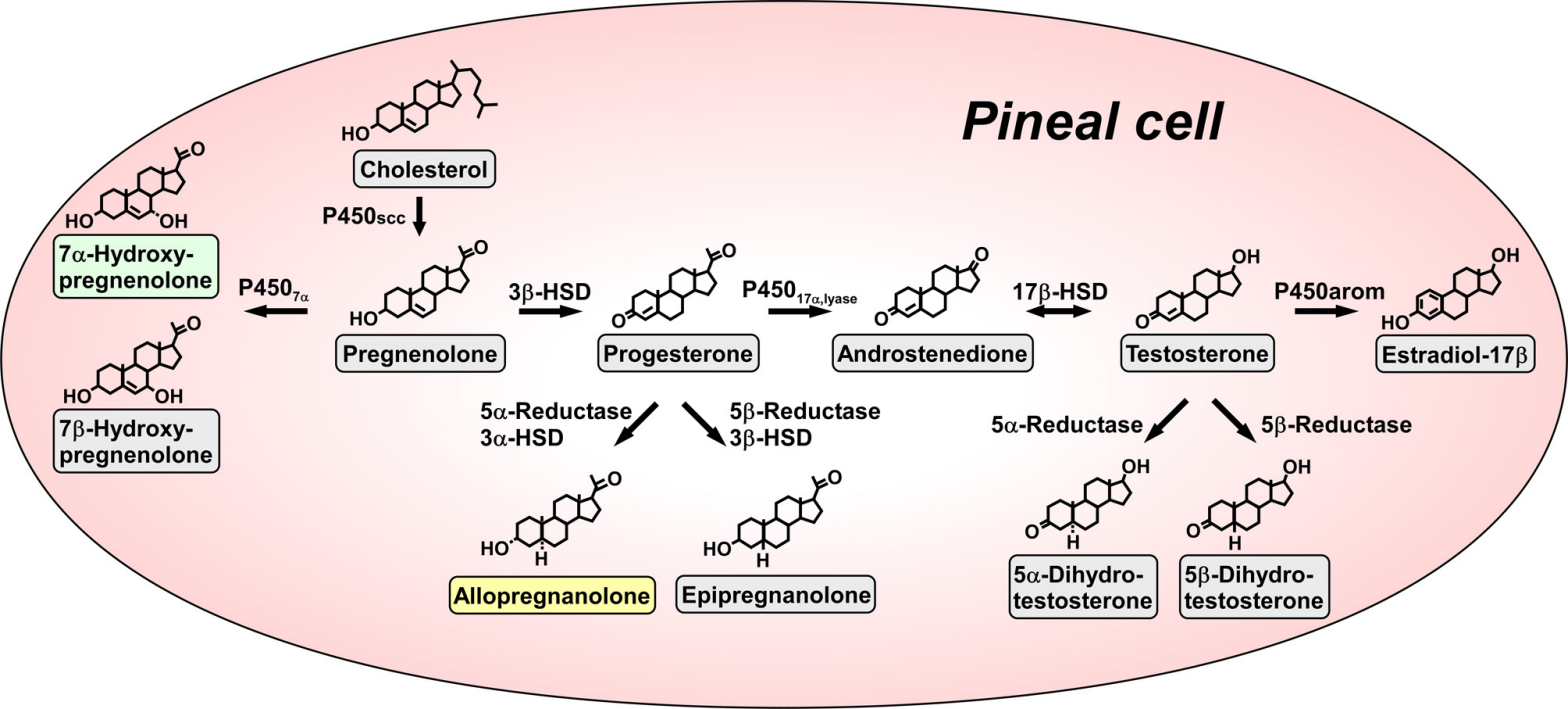
**Biosynthetic pathways for neurosteroids in the pineal gland**. The arrows indicate the biosynthetic pathways of neurosteroids identified in the pineal glands of juvenile quail. The pineal gland actively produces a variety of neurosteroids *de novo* from cholesterol. Allopregnanolone and 7α-hydroxypregnenolone are major products secreted by the pineal gland. P450scc, cytochrome P450 side-chain cleavage enzyme (gene name *Cyp11a*); P450_7α_, cytochrome P450 7α-hydroxylase (gene name *Cyp7b*); 3β-HSD, 3β-hydroxysteroid dehydrogenase/Δ^5^−Δ^4^-isomerase (gene name *Hsd3b*); 3α-HSD, 3α-hydroxysteroid dehydrogenase/Δ^5^−Δ^4^-isomerase (gene name *Hsd3a*); 5α-reductase (gene name *Srd5a*); 5β-reductase (gene name *Srd5b*); P450_17α, lyase_, cytochrome P450 17α-hydroxylase/c17,20-lyase (gene name *Cyp17*); 17β-HSD, 17β-hydroxysteroid dehydrogenase (gene name *Hsd17b*); P450arom, cytochrome P450 aromatase (gene name *Cyp19*). See Haraguchi et al. (2012b) and the text for details.

The pineal gland that is an endocrine organ located close to the parietal region of the brain is known to transduce photoperiodic changes to the neuroendocrine system by rhythmic secretion of melatonin. However, the biosynthesis of neurosteroids in this endocrine organ was, until recently, unknown. We recently provided new evidence that the pineal gland is a major neurosteroidogenic organ actively producing a variety of neurosteroids *de novo* from cholesterol (Hatori et al., 2011; Haraguchi et al., 2012b) (Figure 1). This is a paradigm shift of neurosteroid formation, because it was accepted that neurosteroids are synthesized only in glial cells and neurons which are located in the brain and peripheral nervous systems for the past 30 years (for reviews, see Baulieu, 1997; Tsutsui et al., 1999, 2000, 2003a, 2006; Compagnone and Mellon, 2000; Mellon and Vaudry, 2001; Tsutsui and Mellon, 2006; Do-Rego et al., 2009).

Pregnenolone is a main precursor of steroid hormones and the production of pregnenolone is initiated by cleavage of the cholesterol side-chain by cytochrome P450scc (P450scc; gene name *Cyp11a*), a mitochondrial enzyme, in vertebrates. We first showed that the pineal gland expresses P450scc in juvenile chickens and quail by reverse transcription polymerase chain reaction (RT-PCR) analysis (Hatori et al., 2011; Haraguchi et al., 2012b) (Figure 1). P450scc antibodies stained the cells forming follicular structures in the pineal gland of juvenile birds (Haraguchi et al., 2012b). Incubation of pineal glands from juvenile birds with ^3^H-cholesterol led to the formation of radioactive pregnenolone as revealed by high-performance liquid chromatography (HPLC) analysis (Haraguchi et al., 2012b) (Figure 1). Gas chromatography-mass spectrometry (GC-MS) analysis further demonstrated the occurrence of pregnenolone in the pineal gland (Haraguchi et al., 2012b).

Subsequently, RT-PCR analyses demonstrated the expressions of key steroidogenic enzymes, such as cytochrome P450 7α-hydroxylase (P450_7α;_ gene name *Cyp7b*), 3α-hydroxysteroid dehydrogenase/Δ^5^−Δ^4^-isomerase (3α-HSD; gene name *Hsd3a*), 3β-hydroxysteroid dehydrogenase/Δ^5^−Δ^4^-isomerase (3β-HSD; gene name *Hsd3b*), 5α-reductase (gene name *Srd5a*), 5β-reductase (gene name *Srd5b*), cytochrome P450 17α-hydroxylase/c17,20-lyase (P450_17α, lyase_; gene name *Cyp17*), 17β-hydroxysteroid dehydrogenase (17β-HSD; gene name *Hsd17b*) and cytochrome P450 aromatase (P450arom; gene name *Cyp19*) in the pineal gland of juvenile birds (Hatori et al., 2011; Haraguchi et al., 2012b) (Figure 1).

Biochemical studies combined with HPLC and GC-MS analyses were further conducted to demonstrate the biosynthetic pathways of neurosteroids in the pineal gland. Incubation of pineal glands from juvenile birds with ^3^H-pregnenolone as a precursor led to the formation of 7α- and/or 7β-hydroxypregnenolone as revealed by HPLC analysis (Haraguchi et al., 2012b) (Figure 1). In addition to these neurosteroids, progesterone, allopregnanolone (3α,5α-THP) and/or epipregnanolone (3β,5β-THP), androstenedione, testosterone, 5α- and/or 5β-dihydrotestosterone and estradiol-17β were produced from the precursor pregnenolone (Haraguchi et al., 2012b) (Figure 1). Isomers, such as 7α- and 7β-hydroxypregnenolone; allopregnanolone and epipregnanolone; and 5α- and 5β-dihydrotestosterone, were not separated by HPLC analysis, but GC-MS analysis was capable of separating several pairs of isomers (Haraguchi et al., 2012b). As summarized in Figure 1, pregnenolone, 7α- and 7β-hydroxypregnenolone, progesterone, allopregnanolone, epipregnanolone, androstenedione, testosterone, 5α- and 5β-dihydrotestosterone, and estradiol-17β were identified as the neurosteroids produced in the pineal gland (Haraguchi et al., 2012b). In sum, molecular and biochemical techniques have demonstrated that the pineal gland produces a variety of neurosteroids from cholesterol *via* pregnenolone in juvenile birds. This is the first observation of *de novo* neurosteroidogenesis in the pineal gland in any vertebrate.

### Major pineal neurosteroids

We further investigated major neurosteroids formed and released in the pineal gland. Incubation of the pineal glands from juvenile birds with ^3^H-pregnenolone led primarily to the formation of 7α- and/or 7β-hydroxypregnenolone and allopregnanolone and/or epipregnanolone as revealed by HPLC analysis (Haraguchi et al., 2012b). The formation of 7α- and/or 7β-hydroxypregnenolone and the expression P450_7α_ mRNA in the pineal gland of juveniles were higher than those of adults (Haraguchi et al., 2012b). The formation of allopregnanolone and/or epipregnanolone and the expression of 5α-reductase mRNA in the pineal gland of juveniles were also higher than those of adults (Haraguchi et al., 2012b). Surprisingly, in juvenile birds, the formation of 7α- and/or 7β- hydroxypregnenolone and the expression of P450_7α_ mRNA in the pineal gland were higher than those in brain regions, such as the diencephalon and cerebellum (Haraguchi et al., 2012b). The formation of allopregnanolone and/or epipregnanolone and the expression of 5α-reductase mRNA in the pineal gland were also higher than those in the diencephalon and cerebellum in juvenile birds (Haraguchi et al., 2012b). Thus, the pineal gland of juvenile birds produces 7α- and/or 7β-hydroxypregnenolone and allopregnanolone and/or epipregnanolone far more abundantly than brain tissue.

Subsequently, to clarify the release of neurosteroids from the pineal gland, the pineal glands of juvenile birds were cultured in medium 199. The released neurosteroids were measured by GC-MS. Unlike 7β-hydroxypregnenolone and epipregnanolone, significant amounts of 7α-hydroxypregnenolone and allopregnanolone were released from the pineal gland into the culture medium (Haraguchi et al., 2012b). Thus, it appears that 7α-hydroxypregnenolone and allopregnanolone are the major neurosteroids secreted from the pineal gland (Haraguchi et al., 2012b) (Figure 1).

## Biological action of pineal allopregnanolone on purkinje cell survival during development

**Figure 2:**
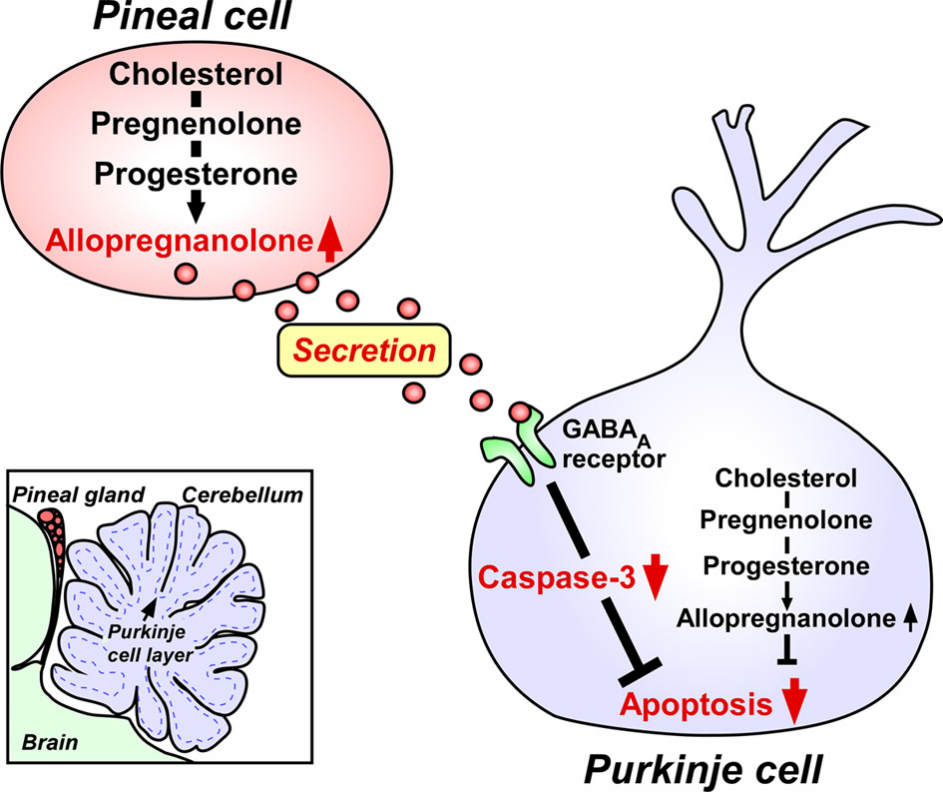
**Neuroprotective action of pineal allopregnanolone on Purkinje cell survival during cerebellar development**. The square in the left bottom indicates the location of the pineal gland in the quail chick brain. The pineal gland is located adjacent to the cerebellum. Allopregnanolone is exceedingly produced in the pineal gland compared with brain regions, and may affect the adjacent cerebellar Purkinje cells by diffusion, and saves Purkinje cells from apoptosis in the juvenile quail. Secreted pineal allopregnanolone inhibits the expression of active caspase-3 that facilitates apoptosis of Purkinje cells in the cerebellum during development. See Haraguchi et al. (2012b) and the text for details.

The two major pineal neurosteroids, 7α-hydroxypregnenolone and allopregnanolone, are abundantly released from the pineal gland during development (Haraguchi et al., 2012b). Therefore, these major pineal neurosteroids may play important roles in the avian brain during development. In birds, the pineal gland is located near the cerebellum (Figure 2). The Purkinje cell integrates the process of memory and learning. It has been reported that, in birds and mammals, pinealectomy (Px) induces cell loss in the brain including Purkinje cells during development (Kilic et al., 2002; Tunç et al., 2006). Based on these findings, we hypothesized that allopregnanolone and/or 7α-hydroxypregnenolone secreted by the pineal gland may play a role in preventing the death of developing Purkinje cells. To test this hypothesis, we conducted a series of experiments in the male juvenile birds. Px decreased the concentration of allopregnanolone in the cerebellum and induced apoptosis of Purkinje cells, whereas administration of allopregnanolone to Px birds increased allopregnanolone concentration in the cerebellum and prevented apoptosis of Purkinje cells (Haraguchi et al., 2012b). We further indicated that pineal allopregnanolone reaches Purkinje cells in the cerebellum by diffusion shown by injection of ^3^H-allopregnanolone close to the pineal lumen (Haraguchi et al., 2012b). Thus, allopregnanolone secreted by the pineal gland is considered to be a key factor for Purkinje cell survival during development (Figure 2).

In contrast to allopregnanolone, administration of 7α-hydroxypregnenolone to Px birds did not increase Purkinje cell survival (Haraguchi et al., 2012b). Although 7α-hydroxypregnenolone did not facilitate Purkinje cell survival, recent studies have demonstrated that this neurosteroid is involved in the regulation of locomotor rhythms of birds (Tsutsui et al., 2008; Hatori et al., 2011).

The induction of cell death of Purkinje cells in the cerebellum by Px suggests that certain other component(s) in the pineal gland may contribute to Purkinje cell survival during development. However, pineal melatonin did not facilitate Purkinje cell survival during development in juvenile birds (Haraguchi et al., 2012b). It thus appears that allopregnanolone but not melatonin acts as an important component of the pineal gland for Purkinje cell survival during development. Allopregnanolone produced in the pineal gland is considered to reach the target site within the cerebellum by diffusion, because allopregnanolone was abundantly released from cultured pineal gland of juvenile birds (Haraguchi et al., 2012b).

## Mode of action of pineal allopregnanolone on purkinje cell survival during development

Finally, we investigated the mode of action of pineal allopregnanolone on Purkinje cell survival. Caspase-3, a crucial mediator of apoptosis, is known to play an important role in Purkinje cell death in vertebrates (Puig and Ferrer, 2001; Matsunaga et al., 2004b; Olkowski et al., 2008). Interestingly, Px increased the number of Purkinje cells that expressed active caspase-3 in juvenile birds and administration of allopregnanolone to Px birds decreased the number of Purkinje cells expressing active caspase-3 (Haraguchi et al., 2012b). Accordingly, the neuroprotective effect of pineal allopregnanolone on Purkinje cells is accompanied with the decrease in caspase-3 activity during development. We thus provide new evidence that pineal allopregnanolone exerts antiapoptotic effects in Purkinje cells by suppressing the activity of caspase-3 during development (Figure 2).

It is unclear whether the action of pineal allopregnanolone on caspase-3 activity in the Purkinje cell is rapid (i.e., mediated through a membrane receptor) or slow (i.e., involving transcriptional activation). On the other hand, the action of allopregnanolone produced in the brain is likely mediated through interaction with the pathway of γ-aminobutyric acid type A (GABA_A_) receptor, since allopregnanolone is a potent allosteric modulator of GABA_A_ receptor (Paul and Purdy, 1992; Lambert et al., 1995). However, the mode of action of pineal allopregnanolone suppressing the activity of caspase-3 in the Purkinje cell remains unclear. We need to clarify the mode of action exerting neuroprotective effect of pineal allopregnanolone in the Purkinje cell.

## Involvement of pineal and brain allopregnanolone in purkinje cell survival during development

The Purkinje cell is known as a major site of neurosteroid formation in the brain of various vertebrates (for reviews, see Tsutsui, 2008a,b). In mammals, the Purkinje cell possesses several kinds of steroidogenic enzymes, such as P450scc and 3β-HSD, and actively produces progesterone during neonatal life (Furukawa et al., 1998; Ukena et al., 1998, 1999) (Figure 2). Allopregnanolone is also synthesized in the neonatal cerebellum (Tsutsui and Ukena, 1999; Tsutsui et al., 2003b,c, 2004; Agís-Balboa et al., 2006, 2007) (Figure 2). Subsequently, biological actions of progesterone (Sakamoto et al., 2001b, 2002, 2003b; Ghoumari et al., 2003) and allopregnanolone (Griffin et al., 2004; Langmade et al., 2006) have been demonstrated by the studies on mammals using the Purkinje cell. In addition, this neuron expresses P450arom, a key enzyme of estrogen formation, and actively produces estradiol-17β in the neonate (Sakamoto et al., 2003a; Tsutsui et al., 2003b). Estradiol-17β also contributes to important events in the developing Purkinje cell (Sakamoto et al., 2003a; Sasahara et al., 2007). Purkinje cells express the receptors for progesterone and estradiol-17β and these neurosteroids promote dendritic growth, spinogenesis, and synaptogenesis of Purkinje cells via each cognate nuclear receptor during cerebellar development (Sakamoto et al., 2001b, 2002, 2003a,b; Sasahara et al., 2007).

It has been shown that allopregnanolone produced in the cerebellum is involved in Purkinje and granule cell survival (Griffin et al., 2004; Langmade et al., 2006) (Figure 2), although allopregnanolone failed to promote dendritic growth, spinogenesis, and synaptogenesis of Purkinje cells (Sakamoto et al., 2001b, 2002). The Niemann–Pick type C (NP-C) mouse has been used as an excellent animal model for understanding the action of allopregnanolone. NP-C is an autosomal recessive, childhood neurodegenerative disease characterized by defective intracellular cholesterol trafficking, resulting in Purkinje cell degeneration as well as neuronal degeneration in other regions. Brains from adult NP-C mice contained less allopregnanolone than wild-type (WT) brain (Griffin et al., 2004). Administration of allopregnanolone to neonatal NP-C mice increased Purkinje cell survival and delayed neurodegeneration (Griffin et al., 2004). According to Langmade et al. (2006), Purkinje cell number was reduced in *npc1*^−/−^ mice, a model of NP-C disease, compared with WT mice. Thus, allopregnanolone produced in the cerebellum acts as a survival factor of Purkinje cells in the neonate (Griffin et al., 2004; Langmade et al., 2006) (Figure 2).

In addition to these findings, our recent studies on juvenile birds have demonstrated that the pineal gland is a major site of production of neurosteroids *de novo* from cholesterol (Hatori et al., 2011; Haraguchi et al., 2012b; Tsutsui et al., 2013a). Notably, allopregnanolone is exceedingly produced in the pineal gland compared with the brain and this major pineal neurosteroid is abundantly released from the pineal gland (Haraguchi et al., 2012b; Tsutsui et al., 2013b,c). Importantly, allopregnanolone secreted by the pineal gland prevents cell death of Purkinje cells by suppressing the activity of caspase-3, a crucial mediator of apoptosis, in the cerebellum during development (Haraguchi et al., 2012b; Tsutsui et al., 2013b,c). Taken together, it appears that both pineal allopregnanolone and cerebellar allopregnanolone are involved in Purkinje cell survival during development (Figure 2).

## Conclusions and future directions

The pineal gland actively produces neurosteroids *de novo* from cholesterol in juvenile birds. This is a new aspect of the biosynthesis of neurosteroids, because it was accepted that neurosteroids are produced only in glial cells and neurons which are located in the brain and peripheral nervous systems. The major pineal neurosteroid allopregnanolone prevents cell death of Purkinje cells by suppressing the activity of caspase-3 during development. P450scc is expressed in the cells forming follicular structures in the pineal gland (Haraguchi et al., 2012b). Further study is needed to determine which cell types within the pineal gland, such as epithelial cells and/or neuronal cells express steroidogenic enzymes, P450scc, 3α- and 3β-HSD, 5α-reductase, *etc*. The coordinated action of steroidogenic enzymes is essential for neurosteroidogenesis. As for the production of allopregnanolone in the pineal gland, the coordinated action of P450scc, 3α- and 3β-HSD and 5α-reductase is required. Therefore, future study is also needed to determine whether all these enzymes are expressed in the same cell. Interaction of pineal and brain allopregnanolone in the regulation of brain development deserves further investigations. Px not only induces cell loss in the brain including Purkinje cells during development (Kilic et al., 2002; Tunç et al., 2006) but also abolishes circadian rhythm of locomotor activity (Gaston and Menaker, 1968; Tsutsui et al., 2008). In addition, allopregnanolone administration increases locomotion (Darbra and Pallarès, 2009). These observations indicate that pineal allopregnanolone may play an important role in the regulation of circadian locomotor activity.

### Conflict of interest statement

The authors declare that the research was conducted in the absence of any commercial or financial relationships that could be construed as a potential conflict of interest.
